# Progress in Medicine

**Published:** 1894-11-10

**Authors:** 


### PROGRESS IN MEDICINE.

### DISEASES OF THE HEART AND VASCULAR SYSTEM.—(*Continued.*)

**Cardiac Asthenia.**—Da Costa[1] describes a form of weak heart that is not clearly recognised yet has features of its own. This form is altogether distinct from those usually described as due to organic lesions, such as fatty degeneration, dilatation, myocarditis, to infectious and diathetic diseases, or to tobacco, alcohol, &c. The heart feebleness to which Da Costa directs attention comes from two causes; it is due either to nervous failure or to a weak heart muscle—in some instances to a combination of both. The affection manifests itself in those whose nervous system has been strained by worry and overwork, and shows itself frequently by a veritable and sudden cardiac collapse, though the causes that have led to this become apparent enough when inquired into, or there have been warning signs. The heart's action is feeble, the pulse very small and compressible and generally increased in frequency, and there is a sense of uneasiness in the cardiac region, but very rarely actual pain. Respiration is not as a rule affected, but there is insomnia, constipation, and a state of mental depression and apprehension. There is no alteration in cardiac dulness, but the impulse is feeble.

Cardiac asthenia, due to weakness of the myocardium is not so common as are cases of nervous origin, and is characterised by dyspnœa on exertion. Those due to affections of the motor ganglia are sudden in onset, and respiration is unaffected.

The treatment consists chiefly in prolonged rest in bed, followed by cold douching, massage, &c. The drugs recommended by Da Costa are strychnine and arsenite of sodium, in doses of $\frac{1}{30}$ grain and $\frac{1}{100}$ of a grain respectively, thrice daily.

Of heart tonics digitalis is best adapted to cases with muscle weakness, but in a number of cases it does not suit at all. Adonidin and chloride of barium have done good service; cactus and convallaria have been disappointing. Caffein and cocaine are both valuable, but their action cannot be kept up. Nitroglycerine is not of much avail, except there be cardiac pain or in combination with remedies like digitalis, which act more distinctly on the force of the heart. Bromides, valerian, and opium ought, in Da Costa's opinion, to be left to meet special indications of nervous disturbance.

**The "Schott" Treatment** of chronic diseases of the heart by baths and exercises has been taken up by Bezley Thorne, who reports most favourably on his results. He remarks that it has been reserved to the Drs. Schott[2] to devise a system by means of which the forces which are brought to bear upon the heart by bodily exercise, and by certain outward applications, in the form of baths, may be regulated in such a way as not only to avoid injury to that organ, but to endow it with such recovery of function and repair of tissue as may constitute it actually or practically sound. The baths most frequently employed in the system are those of Nauheim, containing about 1½ per cent. of chloride of sodium, with about the same amount per thousand of chloride of calcium, salts of iron, a proportion of natural carbonic acid gas made to vary according to the circumstances of the case, and possessing a natural temperature of about 88 deg. to 95 deg. Fahr., but the baths may be prepared artificially, their mineral strength, duration, and frequency all being increased as the treatment proceeds. An interval is imposed after each second, third, or fourth bath taken in daily succession, and it is prescribed that each bath should be followed by an hour's rest in the recumbent position, careful observations being made of the patient's general condition, pulse, and heart, both before and after the baths. The baths should produce cutaneous excitation with a sense of refreshment. Their effect on the pulse is at once to reduce its frequency and to increase its force, and very soon the heart is induced to expel the blood stream faster than it can return. In the case of a patient whose pulse when he was in a state of repose was 78 to 84 a minute, the heart being quite sound, Dr. Thorne found that on four occasions of immersion in one of the effervescing baths it had within two minutes fallen to from 60 to 64, and in ten minutes it had risen to from 66 to 68. The exertion of dressing raised it somewhat, but after walking a quarter of a mile and assumed the recumbent position it fell to from 62 to 68, and thus the influence of the bath not being limited to the period of immersion, it is, he remarks, reasonable to suppose that the allied results are not more transient.

As regards the exercises they consist in movements calculated to bring into successive and regulated action almost every collective system of voluntary muscles. The patient is directed to breathe slowly and regularly during their performance, and to warn the attendant of any approach to palpitation. It is the office of the latter to resist each of the movements prescribed with a measure of force which just falls short of that which would be necessary to arrest it, to impose a short interval between each movement, and carefully to avoid inducing in the patient any indications of distressed breathing. The exercises consist of flexions and extensions, as well as adductions, abductions, and rotations of the extremities, both upper and lower in succession, together with rotations and flexions of the trunk, care being taken that the same movement should not be practised twice in immediate succession. Their effect on the pulse is to materially increase its force, although diminution in the frequency of the beat is often delayed, sometimes even for a few days, but not so the diminution in the size of a dilated heart. Bezley Thorne has observed in one hour the shrinking of the area of dulness, as measured obliquely, of four and even six centimetres, and of two and three centimetres in the vertical line. The exercises as a rule should not be continued beyond half-an-hour at a time. Such diminution is not, of course, at once permanent, the dilated organ tending to return to its former condition in the daily intervals.

Dr. Theodor Schott, as the result of observations during the past eighteen years, affirms that benefit may be expected to accrue in all cases of chronic heart disease, whether of valvular or parietal incidence, except where such conditions have led to or co-exist with, either advanced myocardial or arterio-sclerotic degeneration or aneurism of either the heart wall or great vessel, and Bezley Thorne has himself been a witness of improvement amounting to practical—or more rarely, actual—cure in such cases as the following: Stenosis of either the aortic or mitral orifices; stenosis of both; insufficiency of either or both, in each case with dilatation; dilatation consequent on myocarditis, on habitual hæmorrhage, and on anæmia; fatty degeneration of the heart; weakened heart; congenital mitral insufficiency; potency of the foramen ovale; and angina pectoris of both neurotic and organic causation. He gives notes of several very striking cases, illustrating the therapeutic value of the treatment in cardiac disease.

**Idiopathic Hypertrophy of the Heart.**—S. Laache, at the Rome Congress, discussed this form of enlargement of the heart occurring without obvious anatomical or mechanical obstacles to the circulation while at the same time the valves are intact. He cited as predisposing causes (1) heredity, and (2) those conditions which betray their influences by defective nutrition; while among the determining causes there are two which, above all others, have given rise to discussion during recent years, viz., alcoholism and excessive muscular exercise. The former is of considerable importance, especially when it takes the form of beer drinking, which, according to Bollinger, is the preponderating cause; the form called pure idiopathic hypertrophy arising from plethora due to the ingestion of immoderate quantities of beer and the augmented blood pressure thus produced, combined with a direct injurious action on the heart muscle. The other chief causes are overstrain from excessive athletics or intellectual overpressure.

[1] Amer. Journ. of Med. Sc., p. 361, 1894. [2] Lancet, vol. ii., p. 1,117, 1894.

